# Inhaled β-agonist does not modify sympathetic activity in patients with COPD

**DOI:** 10.1186/s12890-015-0054-7

**Published:** 2015-04-30

**Authors:** Helge Haarmann, Cordula Mohrlang, Uta Tschiesner, David B Rubin, Thore Bornemann, Karin Rüter, Slavtcho Bonev, Tobias Raupach, Gerd Hasenfuß, Stefan Andreas

**Affiliations:** Clinic for Cardiology and Pneumology, University Medical Center Göttingen, Göttingen, Germany; GlaxoSmithKline (GSK), Munich, Germany; Mannheim Biomedical Engineering Laboratories, Medical Faculty at Heidelberg University, Mannheim, Germany; Lung Clinic Immenhausen, Immenhausen, Krs. Kassel, Germany; GlaxoSmithKline, Research Triangle Park, NC, USA

**Keywords:** Chronic obstructive pulmonary disease, Sympathetic activity, Beta agonist, Salmeterol

## Abstract

**Background:**

Neurohumoral activation is present in COPD and might provide a link between pulmonary and systemic effects, especially cardiovascular disease. Because long acting inhaled β-agonists reduce hyperinflation, they could reduce sympathoexcitation by improving the inflation reflex. We aimed to evaluate if inhaled therapy with salmeterol reduces muscle sympathetic nerve activity (MSNA) evaluated by microneurography.

**Methods:**

MSNA, heart rate, blood pressure, and respiration were continually measured. After baseline recording of 20 minutes, placebo was administered; after further 45 minutes salmeterol (50 μg) was administered which was followed by a further 45 minutes of data recording. Additionally, lung function, plasma catecholamine levels, arterial pulse wave velocity, heart rate variability, and baroreflex sensitivity were evaluated. Following 4 weeks of treatment with salmeterol 50 μg twice daily, measurements were repeated without placebo administration.

**Results:**

A total of 32 COPD patients were included. Valid MSNA signals were obtained from 18 patients. Change in MSNA (bursts/100 heart beats) following acute administration of salmeterol did not differ significantly from the change following placebo (-1.96 ± 9.81 vs. -0.65 ± 9.07; p = 0.51) although hyperinflation was significantly reduced. Likewise, no changes in MSNA or catecholamines were observed after 4 weeks. Heart rate increased significantly by 3.8 ± 4.2 (p < 0.01) acutely and 3.9 ± 4.3 bpm (p < 0.01) after 4 weeks. Salmeterol treatment was safe and well tolerated.

**Conclusions:**

By using microneurography as a gold standard to evaluate sympathetic activity we found no change in MSNA following salmeterol inhalation. Thus, despite an attenuation of hyperinflation, the long acting β-agonist salmeterol does not appear to reduce nor incite sympathoexcitation.

**Trial registration:**

This study was registered with the European Clinical Trials Database (EudraCT No. 2011-001581-18) and ClinicalTrials.gov (NCT01536587).

**Electronic supplementary material:**

The online version of this article (doi:10.1186/s12890-015-0054-7) contains supplementary material, which is available to authorized users.

## Background

Patients with chronic obstructive pulmonary disease (COPD) are at increased risk of death and disability from various extra pulmonary complications [1,2]. In COPD patients, cardiovascular diseases are the most common comorbidities [3], are one of the leading causes of hospitalization and one of the main causes of death [4].

It is well known that inhaled β2-agonists induce adverse effects, such as increased heart rate and tremor [5]. This is thought to be due to spillover of the β-agonist into the systemic circulation thereby causing direct activation of β2 and β1 receptors within the cardiovascular system. Furthermore systemic β-agonists (e.g. adrenaline) have excitatory effects on the autonomic nervous system such as direct activation of sympathetic nerve activity [6,7]. Long-acting β2-agonists (LABA) have an acceptable safety profile, although there is still controversy as to whether LABA may increase the risk of asthma mortality [8] or new cardiovascular events in older COPD patients [9].

A striking elevation of sympathetic tone is present in COPD and might provide a link between COPD and systemic effects, specifically cardiovascular disease [10-14]. Several mechanisms related to lung function impairment such as chemoreflexes [15], impaired baroreflexes [16] and hyperinflation [17] contribute to sympathetic activation in COPD [18,19].

In COPD β2-agonists improve lung function, quality of life and exacerbations [5]. Since these drugs reduce bronchoconstriction and hyperinflation, it is conceivable that inhaled β2-agonists reduce sympathetic activation in COPD. Despite their widespread use, the effects of inhaled β2-agonists on sympathetic activity have never been investigated.

Previous investigations have demonstrated that muscle sympathetic nerve activity (MSNA) evaluated by microneurography reflects short-term changes in sympathetic activity, is highly reproducible, and correlates closely with cardiac norepinephrine spillover [20], therefore being considered the gold standard to evaluate sympathetic activity [21].

This study investigates the effects of LABA therapy on lung function and sympathetic activation in COPD. The latter involved microneurographic recordings of MSNA as well as non-invasive assessments of heart rate variability (HRV), catecholamine levels, and spontaneous baroreflexes. Arterial pulse wave velocity (aPWV) is linked to MSNA, is elevated in COPD and indicates risk for cardiovascular disease and mortality. Accordingly aPWV was also assessed.

## Methods

### Study design

This was a partially blinded, single-arm, single centre study in subjects with COPD GOLD stage II or III. The study included a screening visit and two study visits (visit 1 at week 0 and visit 2 at week 4), and one telephone contact at week 2. At the screening visit, eligibility of the subjects was assessed and written informed consent obtained. At visit 1 a complex measurement period with continuous recordings of MSNA, blood pressure, tidal volume, respiratory rate, oxygen saturation and transcutaneous CO_2_ were conducted while placebo and 50 μg salmeterol were inhaled by the subjects in a sequential manner. Following visit 1, all subjects were treated with salmeterol 50 μg twice daily for 4 weeks. At visit 2, these observations were repeated without placebo inhalation. The subjects were recruited in a single study centre with the help of two local chest physicians and newspaper advertisements.

Male or female patients aged between 40 and 80 years were eligible to enter the study. A prerequisite for inclusion was COPD GOLD class II or III with a post-bronchodilator spirometry forced expiratory volume in one second (FEV_1_) <70% predicted, and current or prior history of ≥10 pack-years of cigarette smoking. The main exclusion criteria were concomitant active lung diseases other than COPD, exacerbation or hospitalisation due to COPD six weeks prior to screening, frequent exacerbations necessitating therapy with inhaled glucocorticosteroids, non-invasive ventilation, treatment with drugs affecting sympathetic tone (e.g. theophylline), use of systemic glucocorticoids or antibiotics, uncontrolled sleep apnoea syndrome, left heart failure with a left ventricular ejection fraction <40%, polyneuropathy, or insulin dependent diabetes mellitus.

The study was performed in a partially blinded manner: The patient was blinded to the sequence of study drug administration (i.e., to the fact that lactose was administered as placebo before salmeterol) at visit 1. Furthermore, the researchers evaluating the recorded data were blinded to the subject, visit and intervention. This was done by pseudonymisation of the recorded data sets of each of the five measurement periods (baseline, placebo, salmeterol at visit 1; baseline and salmeterol at visit 2) and removing the time stamps from each recorded file.

### Study protocol

Subjects were studied two hours after a low-calorie meal free from caffeine-containing beverages. They had been instructed not to smoke any cigarettes for 8 hours before the clinic visit. Study participants were requested to take their usual medication except for diuretics which were to be withheld on the morning of the study visit. The 2-hour measurement period comprised the continuous recording of MSNA, ECG, blood pressure, respiration, oxygen saturation, and transcutaneous CO_2_ with the patient lying in a supine position. About 20 minutes after a stable MSNA signal had been obtained (‘baseline’), one dose of placebo was administered; after a further recording period of 45 minutes one dose of salmeterol (50 μg) was administered which was followed by a further 45 minutes of data recording. We analyzed the last 10 minutes of each of these recording sections (baseline, placebo, salmeterol).

### Muscle sympathetic nerve activity

Sympathetic tone was measured using microneurographic recordings of efferent muscle sympathetic nerve activity in the peroneal nerve [22]. A tungsten microelectrode (shaft diameter 200 μm, tip diameter 1-5 μm, FHC, Bowdoin, USA) was inserted into the nerve. The signal was amplified and integrated (662C-4 Nerve Traffic Analysis System, Absolute Design and Manufacturing Services, Iowa, USA). Intraobserver variation in identifying bursts of efferent sympathetic nerve activity was ~5% in previous studies done in our laboratory [15,23].

### Hemodynamics, baroreflex sensitivity, and heart rate variability

ECG (Datex AS/3, Datex Ohmeda, WI, USA) and continuous blood pressure measurements (Portapres, Finapres Medical Systems, Amsterdam, Netherlands) were used to obtain data from which heart rate variability (HRV) and baroreflex sensitivity (BRS) were computed [24,25].

### Catecholamine and BNP measurements

During MSNA measurement, blood samples of plasma norepinephrine, epinephrine and brain natriuretic peptide (BNP) were taken at least 20 minutes after insertion of an intravenous line into an antecubital vein at baseline, after placebo and after salmeterol inhalation.

### Lung function, pulse wave velocity, and echocardiography

Lung function was assessed before and after the MSNA measurement at visit 1 and visit 2 (at least 60 min. after salmeterol inhalation) via body plethysmography and helium dilution in the sitting position. Carotid-femoral aPWV was evaluated via SphygmoCor (AT Cor Medical, Itasca, USA) before MSNA measurement at visit 1 and visit 2.

Transthoracic echocardiography was performed before visit 1 and visit 2. Because patients need to be completely relaxed during MSNA recordings, lung function tests, aPWV and echocardiography could not be performed during these measurements.

### Sample size calculation

The primary endpoint was the difference in MSNA change elicited by either salmeterol or placebo inhalation (compared to baseline MSNA levels) on visit 1. According to a sample size calculation using data from previous studies (standard deviation of intra-individual difference in MSNA between 9 and 10 bursts/100 heart beats following interventions such as oxygen administration or slow breathing [16,23]), 24 subjects needed to be investigated in order to be able to detect a 10% difference in MSNA change at a significance level of 5% and with 80% power. Under the assumption that adequate microneurographic recordings would only be obtained in 75% of all subjects studied in our lab, 32 subjects needed to be enrolled.

### Statistical analysis

The statistical analysis was performed using SAS-software, version 9.2 (SAS institute, Cary, North Carolina, USA). Data are presented as percentages or means ± standard deviations, as appropriate. Changes in continuous variables were assessed using paired t-tests, and Pearson’s coefficient was calculated for correlations. The significance level was set to 5% (two-sided tests). The safety analysis was based on the analysis of adverse events (AEs).

### Clinical trial registration

The study was registered with BfArM, the European Clinical Trials Database (EudraCT No. 2011-001581-18) and www.clinicaltrials.gov (NCT01536587) and approved by the Ethics Committee of Göttingen University (Application number 11/12/11).

More information on the methods of this study is available as supplementary material (see Additional file 1).

## Results

A total of 32 subjects were enrolled. Two subjects discontinued the study prematurely due to an adverse event (nasopharyngitis and dyspnea; bronchitis).

Demographic and baseline characteristics are summarized in Table 1. Concomitant diseases were reported in 30 subjects (93.8%). Most common were cardiovascular disorders (17 subjects, 53.1%) with hypertension being the condition most frequently reported. Concomitant non-COPD medications were reported in 28 subjects (87.5%). Most common were anti-thrombotic agents (15 subjects, 46.9%), and medications for cardiovascular disease (14 subjects, 43.8%).

**Table 1:** **Demographic and baseline characteristics**

**Gender**	Male, n (%)	21 (65.6)
	Female, n (%)	11 (34.4)
**Age** [years]		61.2 ± 8.4
**Body weight** [kg]		81.5 ± 19.8
**Height** [cm]		174.6 ± 7.6
**Body mass index** [kg/m^2^]		26.5 ± 5.2
**First diagnosis of COPD** [months before screening)		59.0 ± 62.3
**GOLD stage**	II (moderate COPD), n (%)	14 (45.2)
	III (severe COPD), n (%)	17 (54.8)
**COPD symptoms**	Breathlessness, n (%)	31 (96.9)
	Cough, n (%)	24 (75.0)
	Sputum production, n (%)	21 (65.6)
**FEV**_**1**_ [L]		1.6 ± 0.4
**FEV**_**1**_ [% predicted]		50.2 ± 9.9
**FVC** [L]		2.9 ± 0.8
**FEV**_**1**_ **/FVC** [%]		54.5 ± 9.2
**Smoking status**	Previous smoker, n (%)	13 (40.6)
	Smoker, n (%)	19 (59.4)
**Cessation of smoking** [months before screening]		54.0 ± 47.4
**Pack years**		43.1 ± 17.7

COPD = Chronic obstructive pulmonary disease; FEV_1_ = forced expiratory volume in one second; FVC = forced vital capacity; GOLD = Global Initiative for Chronic Obstructive Lung Disease. Values are expressed as mean ± SD unless otherwise stated.

### Medication adherence

As calculated on the basis of the residual doses in the returned dose dispensers 97.5 ± 9.0% of the medication was used by the patients. Use of rescue medication between weeks 2 and 4 was reported by 23 subjects (74.2%, N = 31) at visit 2.

### Muscle sympathetic nerve activity

A valid MSNA signal for visit 1 was obtained in 18 patients. In 14 patients a valid MSNA signal could either not be obtained or was lost in the course of the examination. Intraobserver variation in identifying bursts was 4.01 ± 0.02%.

Baseline MSNA was 74.4 ± 16.3 bursts/100 heart beats. This was higher as compared to historical controls [16] of similar age and BMI. No significant effect of smoking status on baseline MSNA was observed (smokers: 75.91 ± 15.44 vs. non-smokers: 71.58 ± 15.20 bursts/100 heart beats; p = 0.54).

**Figure 1:**
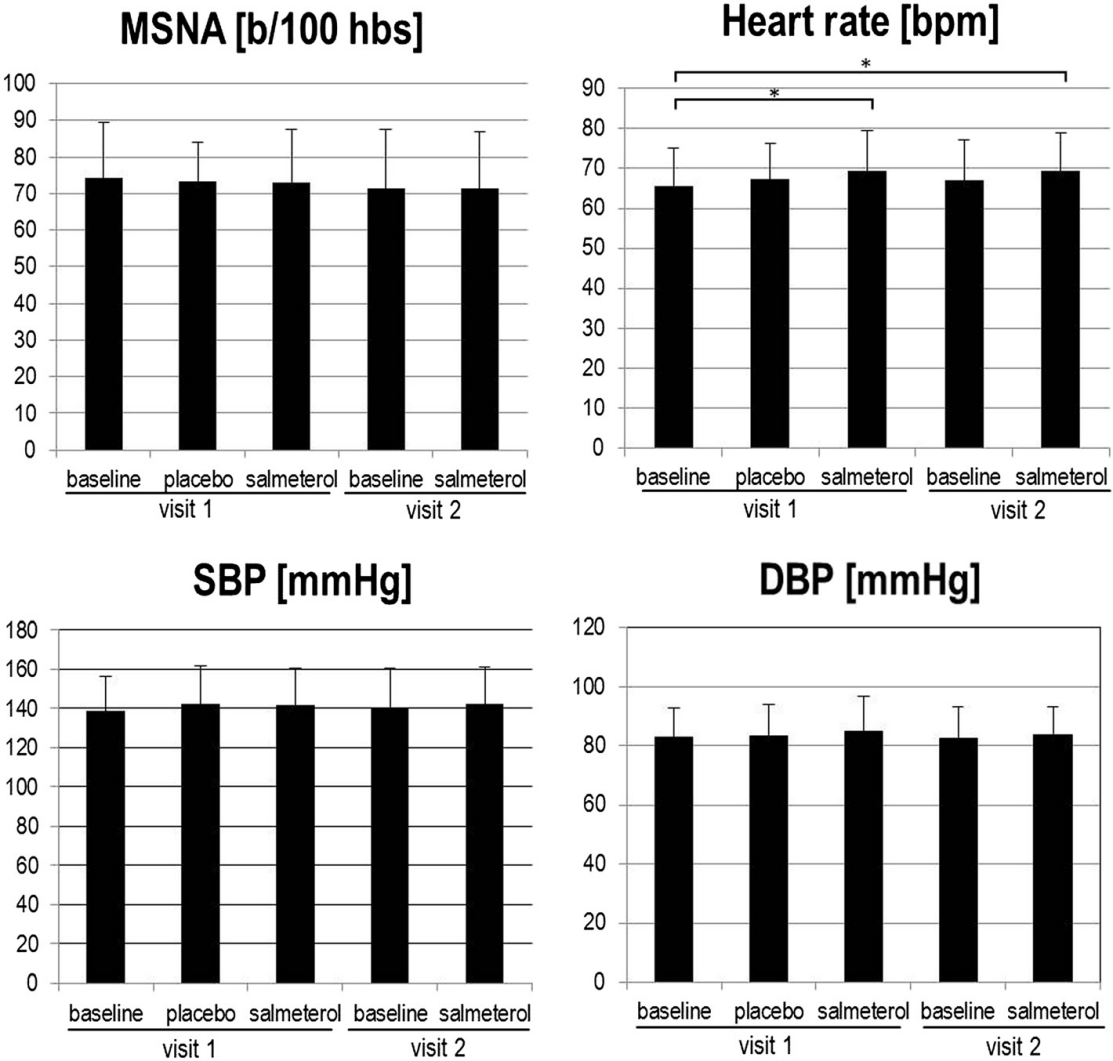
Effects of salmeterol on MSNA, heart rate, systolic and diastolic blood pressure. Displayed are means ± SD. SBP = systolic blood pressure; DBP = diastolic blood pressure. * = significant changes in paired *t*-test (p < 0.05). For further information see results and Table 2.

As shown in Figure 1, both placebo and salmeterol inhalation elicited minor changes in MSNA compared to baseline levels. The change in MSNA (bursts/100 heart beats) following acute administration of salmeterol did not differ significantly from the change following placebo (-1.96 ± 9.81 vs. -0.65 ± 9.07; p = 0.51). MSNA after salmeterol inhalation at visit 2 was not significantly different from MSNA at baseline at visit 1 (change by -3.07 ± 11.59 bursts/100 heart beats; p = 0.38, n = 12).

### Hemodynamics, baroreflex sensitivity, heart rate variability, ventilation and pulse wave velocity

Systolic and diastolic blood pressure as well as BRS showed no relevant changes (Figure 1; Table 2). Heart rate was significantly increased after acute salmeterol inhalation at visit 1 and visit 2 as compared to baseline (Figure 1; Table 2). Resting heart rate after 4 weeks of salmeterol treatment increased from 65.6 ± 9.4 at baseline to 67.1 ± 9.9 bpm at visit 2 (before the administration of salmeterol; p = 0.04 in paired *t*-test). As an acute effect of salmeterol, low frequency power (LF) of HRV, reflecting sympathetic tone, was significantly elevated while high frequency power (HF), reflecting parasympathetic tone, decreased. There was no correlation between changes in heart rate, HRV, BRS and changes in MSNA.

**Table 2:** **Heart rate variability and baroreflex sensitivity**

	**Heart rate [beats/min]**	**SDNN [ms]**	**RMSSD [ms]**	**LF [normalized units]**	**HF [normalized units]**	**Baroreflex sensitivity [ms/mmHg]**
**Visit 1 (Week 0)**	**Mean ± SD; p (two-sided)**					
Baseline	65.6 ± 9.4	59.4 ± 38.8	64.5 ± 61.9	53.6 ± 21.8	46.4 ± 21.8	6.3 ± 3.7
After placebo	67.2 ± 9.0	66.9 ± 48.1	75.1 ± 81.6	52.9 ± 22.2	47.1 ± 22.2	6.8 ± 5.9
After salmeterol	69.4 ± 10.1	57.6 ± 33.2	53.0 ± 49.4	62.1 ± 20.3	37.9 ± 20.3	6.2 ± 3.9
Change ‘after salmeterol’ – Baseline	3.7 ± 4.2; p = <0.01	-1.7 ± 26.4; p = 0.71	-11.5 ± 36.3; p = 0.08	8.4 ± 14.5; p < 0.01	-8.4 ± 14.5; p < 0.01	0.1 ± 1.9; p = 0.84
**Visit 2 (Week 4)**						
Before salmeterol	67.1 ± 9.9	57.0 ± 43.3	55.4 ± 70.4	59.9 ± 19.0	40.1 ± 19.0	7.7 ± 5.1
After salmeterol	69.4 ± 9.4	60.2 ± 48.8	60.8 ± 84.4	55.3 ± 22.8	44.7 ± 22.8	6.5 ± 5.2
Change ‘after salmeterol’ – Baseline	3.9 ± 4.3; p < 0.01	0.1 ± 53.9; p = 0.99	-5.2 ± 96.1; p = 0.76	2.5 ± 23.3; p = 0.55	-2.5 ± 23.3; p = 0.55	0.2 ± 2.6; p = 0.62

Baseline = value at visit 1 before any inhalation; HF = High frequency component; LF = Low frequency component; SDNN = Standard deviation of the NN interval; RMSSD = Root mean square of the successive differences.

The course of lung function and respiratory parameters is shown in Table 3. Mean tidal volume and mean respiratory minute volume increased after salmeterol inhalation at visit 1. This effect could not be observed after 4 weeks of salmeterol treatment. TLC, RV and FRC significantly decreased after acute and long-term salmeterol inhalation, while FEV_1_ was unchanged. Capillary blood gas analysis (Table 4), continuous measurements of oxygen saturation and transcutaneous pCO_2_ revealed no relevant changes.

**Table 3:** **Lung function and ventilation**

	**FEV**_**1**_ **[L]**	**FVC [L]**	**FRC [L]**	**FRC (helium dilution) [L]**	**TLC [L]**	**RV [L]**	**Respiratory rate [/min]**	**Tidal volume [mL]**	**Respiratory minute volume [mL/min]**
**Visit 1 (Week 0)**	**Mean ± SD; p (two-sided)**								
Baseline	1.35 ± 0.46	2.60 ± 0.69	4.65 ± 1.33	3.64 ± 0.97	6.61 ± 1.39	3.90 ± 1.28	15.66 ± 3.34	549 ± 200	8362 ± 3333
After salmeterol	1.31 ± 0.40	2.58 ± 0.62	3.86 ± 1.68	3.21 ± 0.78	5.88 ± 1.89	3.16 ± 1.64	15.17 ± 3.45	610 ± 250;	8934 ± 3942
Change ‘after salmeterol’ – Baseline	-0.04 ± 0.23; p = 0.35	-0.02 ± 0.35; p = 0.72	-0.90 ± 1.66; p < 0.01	-0.52 ± 0.73; p < 0.01	-0.82 ± 1.71; p = 0.01	-0.85 ± 1.79; p = 0.01	-0.49 ± 1.62; p = 0.09	60 ± 118; p = 0.01	572 ± 1530; p = 0.05
**Visit 2 (Week 4)**									
Before salmeterol	1.38 ± 0.44	2.68 ± 0.64	4.20 ± 1.61	3.57 ± 0.86	6.23 ± 1.70	3.46 ± 1.61	15.76 ± 3.74	564 ± 197	8773 ± 3369
After salmeterol	1.32 ± 0.46	2.64 ± 0.71	3.67 ± 1.80	3.38 ± 0.77	5.67 ± 1.83	2.90 ± 1.79	15.48 ± 4.02	593 ± 217	8829 ± 3629
Change ‘after salmeterol’ – Baseline	-0.04 ± 0.21; p = 0.33	0.04 ± 0.35; p = 0.51	-1.26 ± 1.75; p < 0.01	-0.27 ± 0.83; p = 0.18	-1.17 ± 1.84; p < 0.01	-1.30 ± 1.80; p < 0.01	-0.06 ± 2.63; p = 0.90	56 ± 184; p = 0.15	671 ± 2963; p = 0.28

Baseline = value at visit 1 before any inhalation; FVC = Forced vital capacity; FRC = Functional residual capacity; TLC = Total lung capacity; RV = Residual volume.

**Table 4:** **Capillary blood gas analysis**

	**pH**	**SO2c [%]**	**pCO2 [mmHg]**	**pO2 [mmHg]**
	**Mean ± SD; p (two-sided)**			
**visit 1** **(Week 0)**	7.43 ± 0.03	92.06 ± 4.79	39.56 ± 3.77	65.69 ± 10.56
**visit 2** **(Week 4)**	7.43 ± 0.03	92.10 ± 4.80	39.26 ± 2.98	65.10 ± 9.83
**Change visit 2** **– visit 1**	-0.002 ± 0.02; p = 0.61	0.03 ± 2.60; p = 0.95	-0.29 ± 2.95; p = 0.59	-0.68 ± 9.39; p = 0.69

SaO2 = oxygen saturation; pCO2 = carbon dioxide tension; pO2 = oxygen tension.

Echocardiographic data (not shown) and carotid-femoral aPWV was unchanged after 4 weeks of salmeterol treatment (9.17 ± 2.06 vs. 8.83 ± 2.23 m/s; p = 0.39).

### Catecholamine and BNP measurements

**Figure 2:**
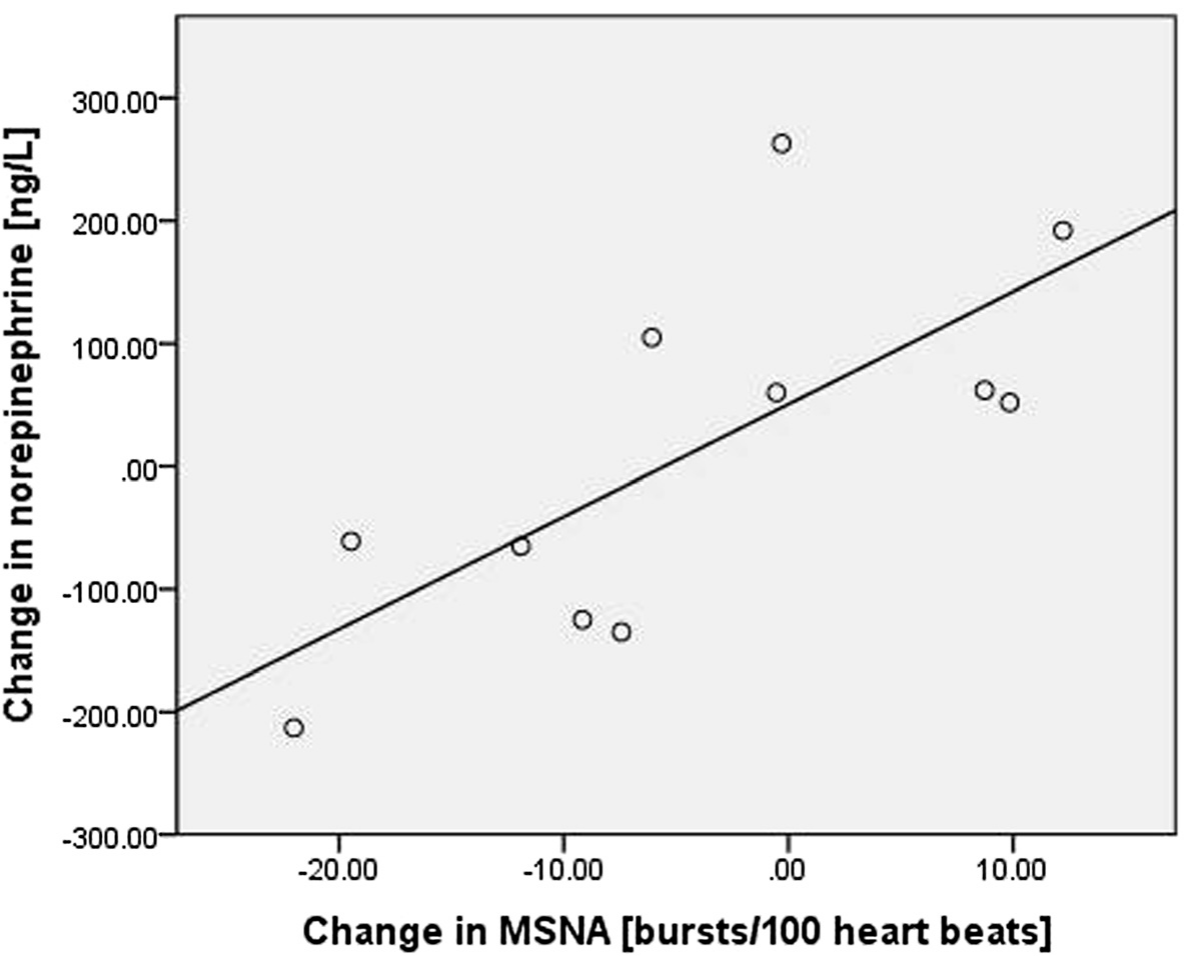
Scatter plot of change in norepinephrine against change in MSNA. Displayed are changes from visit 1 (week 0) to after salmeterol inhalation at visit 2 (week 4). (r = 0.72; p = 0.01; n = 11).

The levels of plasma norepinephrine, epinephrine and brain natriuretic peptide did not change following salmeterol inhalation as compared to placebo. However, there was a significant correlation between changes in plasma norepinephrine levels and changes in MSNA after salmeterol administration at visit 2 as compared to visit 1 (r = 0.72; p = 0.01; Figure 2).

## Discussion

For the first time the effect of an inhaled β2-agonist on MSNA was evaluated. The primary endpoint, a significant difference in MSNA change following salmeterol inhalation versus placebo inhalation was not met. However, MSNA and other measures of neurohumoral activation such as plasma norepinephrine, BRS and aPWV did not change following acute or long-term salmeterol inhalation. These findings are of interest, since previous work in patients with heart failure indicates that neurohumoral activation and sympathoexcitation are linked to mortality [26-28].

### Effects on heart rate

Our results showing an increase in heart rate of about 4 beats/minute following inhalation of a sympatho-mimetic drug corroborate older studies. Cazzola and colleagues found an increase in heart rate of about 4 beats/minute with salmeterol in COPD patients with cardiovascular disease [12]. Blood pressure was not evaluated in that study. In a recent study using inhaled fluticasone and the long-acting β2-agonist vilanterol in asthma patients, heart rate was acutely increased by 3.4 beats/minute. Long-term use yielded a decrease in heart rate that was greater with placebo [29]. In COPD patients, the same combination had no effect on heart rate or blood pressure [30]. Similarly, the long acting β2-agonist indacaterol at standard doses had no effect on heart rate or blood pressure in COPD patients [31]. The fact that salmeterol treatment was associated with an increase in heart rate without an increase in MSNA in our study suggests that the increase in heart rate is caused by activation of cardiac β-receptors rather than efferent excitatory traffic of the autonomic nervous system.

Increased resting heart rate is related to mortality in patients with COPD [32] and heart failure [33]. Salmeterol as well as other LABA have been successfully used for inhalation therapy in large studies and for a long time [34]. In controlled randomised trials, β2-agonists improve lung function, quality of live and exacerbations in COPD, but a reduction in mortality was never demonstrated [5]. Interestingly, among older COPD patients, starting LABA treatment was associated with an increased risk of cardiovascular events in a recent nested case control analysis [9].

### Sympathetic activation

MSNA was increased in our patients and as high or even higher as compared to previous studies in COPD patients [16,35]. In a setting of increased MSNA, previous interventional studies using oxygen or slow breathing demonstrated a significant decrease in MSNA [16,35]. However, in the present study no effect of the intervention on MSNA was noted. For more insight into the effects of inhaled β-agonists on sympathetic activity it is helpful to discuss older work on intravenous administration of catecholamines. Adrenaline infusion (with α1-receptor activity causing vasoconstriction and β-receptor activity) did cause an increase in MSNA [6]. Intravenous dobutamine (a predominant β1-agonist and weak β2-agonist without relevant α1 activity) did not increase MSNA in heart failure patients or matched controls [36]. While there was no effect on heart rate or blood pressure in heart failure patients, healthy matched controls experienced an increase in heart rate but not blood pressure. In contrast, in an unmatched group of younger healthy subjects blood pressure increased, and consecutively MSNA decreased following dobutamine infusion [36]. In another study intravenous dobutamine reduced cardiac norepinephrine spillover in patients with heart failure but not in subjects with normal ejection fraction [37]. Similarly, in healthy young subjects the increase in heart rate following salmeterol was explained mainly by a decrease in total peripheral resistance [38,39]. Our study contributes to the understanding of these data by the comprehensive evaluation of respiration, cardiovascular function and MSNA: In our older patients with increased sympathetic activity we did not observe a change in blood pressure or MSNA following salmeterol.

The observed changes in HRV are suggestive of a decrease in parasympathetic tone. Similar results were observed in a small study using the SABA salbutamol [38]. However, given the absence of significant changes in MSNA and baroreflex sensitivity, the HRV findings are difficult to interpret [40]. Furthermore, changes in ventilation and heart rate as present in our study impact on HRV thus rendering interpretation even more problematic.

Sympathoexcitation in COPD is thought to be caused by several mechanisms such as hypoxia, chemoreflexes [15], impaired baroreflexes [16] and lung hyperinflation [17,18]. Lung hyperinflation affects the autonomic nervous system by activation of intrapulmonary non-myelinated afferents and by increased end-expiratory pressure with consecutive impaired left ventricular filling and reduced stroke volume [17,41,42]. Furthermore hyperinflation increases the work of breathing [43] and thus impacts on muscle metaboreceptors [4,16,44]. Thus we speculated that the improvement of lung function and hyperinflation following inhaled therapy reduces sympathetic activation. However, despite reducing hyperinflation, salmeterol did not impact on sympathetic tone in our study. It is possible that the positive effects mediated via bronchiodilatation were too small to elicit a reduction in sympathetic activity. It is also possible, that any positive effect on sympathetic activity were offset by consequences of systemic β-receptor activation. Our study is not able to further dissect these possibilities. In this context it would be interesting to investigate an inhaled therapy with stronger bronchodilator activity.

We studied not only the acute but also the long-term effects of salmeterol over one month. As in the acute setting, MSNA and other measures of autonomic nervous system activity were unchanged. Desensitization or saturation of β2-adrenoceptors that follows regular use of β2-agonist treatment is believed to be responsible for the resolution of hemodynamic findings after the first days and weeks and may also prevent long-term effects of salmeterol on MSNA [5]. However, in our study the acute effects were comparable to the effects after 4 weeks. Specifically the increase in heart rate was similar in the acute and long-term setting.

### Strengths and limitations

In this study, COPD patients underwent a comprehensive protocol including evaluation of the cardiovascular, respiratory and autonomic nervous system. We used microneurography as the gold standard to measure sympathetic activity. The good internal validity of our data is demonstrated by the positive correlation between the change in MSNA and the change in plasma norepinephrine levels. The calculated sample size of 24 patients for the primary endpoint was missed by 6 subjects (n = 18), as we overestimated the rate of successful MSNA registrations. The per visit success rate of MSNA recordings is reported as 70% in literature [45]. We had a 56% success rate on visit 1. The difficulty to keep a stable MSNA signal for a data registration period of almost two hours was underestimated, as previous studies [15,16] had distinct shorter registration periods. As a consequence, the power of the study was slightly decreased.

Although we implemented a placebo application, no parallel design was used. Thus we cannot control for time effects. Nevertheless it is unlikely that the increase in heart rate with constant MSNA and baroreflexes as main findings can be explained by the effect of time. A double blind protocol was not feasible. However, blinded investigators performed evaluation of MSNA, heart rate, HRV, BRS, tidal volume and respiratory rate. In our study salmeterol was a weak bronchodilator because measures of hyperinflation but not FEV_1_ were improved. This finding may be explained by the supine position the patients had to maintain during evaluation of MSNA and that deep inhalation of the study drug is difficult in the supine position. Some other studies were also unable to detect an increase in FEV_1_ with salmeterol despite positive findings concerning hyperinflation or exercise physiology [46].

## Conclusions

By using microneurography as a gold standard to evaluate sympathetic activity we found no change in MSNA following acute or chronic salmeterol inhalation. Thus a relevant negative effect of LABA inhalation on sympathetic activation is highly unlikely. Further studies with stronger bronchodilators are warranted.

## Additional file

Additional file 1:
**Supplementary information on the methods of the study.**

## Abbreviations

BNP: Brain natriuretic peptide
BRS: Baroreflex sensitivity
COPD: Chronic obstructive pulmonary disease
ECG: Electrocardiogram
FEV_1_: Forced expiratory volume in 1 second
FRC: Functional residual capacity
FVC: Forced vital capacity
GSK: GlaxoSmithKline
GOLD: Global Initiative for chronic obstructive lung disease
HRV: Heart rate variability
LABA: Long-acting beta-agonist
MSNA: Muscle sympathetic nerve activity
paCO_2_: Arterial carbon dioxide tension
RV: Residual volume
SABA: Short-acting beta-agonist
SaO2: Arterial oxygen saturation
tCO_2_: Transcutaneous carbon dioxide
TLC: Total lung capacity
VC: Vital capacity
