# Supplementary material for: Inhaled β-agonist does not modify sympathetic activity in patients with COPD
Source: BMC Pulm Med. 2015 Apr 30;15:46. doi: 10.1186/s12890-015-0054-7 (PMC4460951; doi:10.1186/s12890-015-0054-7)
Supplement: Additional file 1: — Supplementary information on the methods of the study. [file 12890_2015_54_MOESM1_ESM.docx]

**“Inhaled β-agonist does not modify sympathetic activity in patients with COPD”**

**Supplementary information on the methods of the study**

**Study design**

A design with sequential inhalation of salmeterol following placebo was applied because microneurography can only be performed for about 2 hours; thereafter the MSNA signal is likely to deteriorate. However, the effects of salmeterol are present much longer than 2 hours making the reverse order and thus a cross-over design impracticable. It was also not feasible to perform a second MSNA registration just following the first, since there is a risk of damaging the peroneal nerve if the second registration is done too early.

**Medication use**

Patients had to withhold tiotropium bromide for 7 days, any LABAs for 48 hours and short-acting beta-agonists (SABA) for 6 hours before visit 1. The investigational product for this study was salmeterol dry powder 50 μg applied via *Diskus®* inhaler. Study treatment started at visit 1. During the subsequent 4-week treatment phase one dose of 50 μg salmeterol was inhaled twice daily. The short-acting beta-agonists fenoterol or salbutamol were used as rescue therapy. Patients had to withhold salmeterol 12 hours and SABA 6 hours before visit 2.

**Secondary endpoints**

Secondary endpoints were changes in HRV, BRS, plasma norepinephrine, epinephrine and BNP levels, aPWV, respiration, lung function, and safety from baseline after acute administration of salmeterol and after 4 weeks of salmeterol treatment. In order to analyse the coherence of our data the correlation between change in MSNA and change in norepinephrine between visit 1 and visit 2 was assessed.

**Monitoring of respiratory parameters**

During MSNA assessment, respiration was continuously monitored through measures of respiratory rate and tidal volume by respiratory inductive plethysmography (Respitrace Systems, Ambulatory Monitoring Inc., New York, USA) [[1](#_ENREF_1)], carbon dioxide tension by transcutaneous sensor (TCM 3, Radiometer, Copenhagen, Danmark) and oxygen saturation via pulse oximetry (Datex AS/3, Datex Ohmeda, WI, USA).

**Data recording and analysis**

Data were recorded and analysed using a multi-channel analogue to digital (A/D) converter (BEM, Mannheim Biomedical Engineering Laboratories, Mannheim, Germany) and compatible software (MedIS, Clinic for Anaesthesiology, University Medical Center Göttingen, Germany; Matlab, MathWorks, Ismaning, Germany).

**References**

1. Raupach, T., et al., *Slow breathing reduces sympathoexcitation in COPD.* Eur Respir J, 2008. **32**(2): p. 387-92.
